# Risk Factors for Interpersonal Violence in Prison: Evidence From Longitudinal Administrative Prison Data in Northern Ireland

**DOI:** 10.1177/08862605211006363

**Published:** 2021-04-13

**Authors:** Michelle Butler, Catherine B. McNamee, Dominic Kelly

**Affiliations:** 1 Queen’s University Belfast, UK; 2 Northern Ireland Prison Service, Belfast, UK

**Keywords:** criminology, violent offenders, cultural contexts

## Abstract

The present study uses a prospective longitudinal research design to examine whether previously identified risk factors for prison interpersonal violence can predict violent prison misconduct in Northern Ireland (NI). Administrative data drawn from the records of 429 adult males imprisoned on November 22, 2017 were used to predict involvement in violent prison misconduct during a 1-year follow-up period. The results revealed that only a small number of previously identified risk factors were found to be significant in the NI context. Nationality, neighborhood deprivation, history of addiction, submission of prison complaints, past involvement in prison misconduct, and number of incarcerations emerged as significant, while religion, head injury/epilepsy, property offences, and prison visits were significant at the marginal level. Given the variation in risk factors identified as significant in the NI context compared to previous research, it is argued that cultural context matters when attempting to generalize the risk factors for prison interpersonal violence from one jurisdiction to another. These results offer some support for the importation theory, although it should be noted that the inclusion of prison environmental factors was limited due to the nature of the data. It is argued that specialist services and supports should be provided to address the factors contributing to interpersonal prison violence, including interventions to improve feelings of fairness, identify and treat underlying medical issues, as well as support visitation.

## Introduction

Efforts to understand the factors influencing prison interpersonal violence have been ongoing for many years, as researchers seek to make prisons safer for those imprisoned and staff (e.g., Bottoms, 1999; Cunningham et al., 2011; Edgar et al., 2003; Rocheleau, 2013; van der Laan & Eichelsheim 2013), as well as lessen the potential for violence to occur in society (e.g., Cochran et al., 2014). Moreover, the knowledge gained from identifying the causes of prison interpersonal violence can inform the development of risk assessments, programs, and interventions seeking to reduce violence (Bottoms, 1999; Cunningham et al., 2008; Edgar et al., 2003). To date, most studies have been conducted in North America, using a cross-sectional or retrospective longitudinal research design (e.g., Cunningham et al., 2011; Gaes et al. 2002; Jiang & Fisher-Giorlando, 2002; Kuanliang et al., 2008; Woo et al., 2020). While this work has been hugely beneficial in identifying the potential causes of prison interpersonal violence, the sparsity of studies conducted outside of North America may impede the generalizability of these findings to other areas (Lai, 2019). In addition, cross-sectional and retrospective longitudinal research designs can make it difficult to determine the temporal relationship between the factors identified and prison interpersonal violence.

This paper begins to address these limitations by examining the factors associated with prison interpersonal violence in Northern Ireland (NI) using a prospective longitudinal research design. In this way, the article contributes to new knowledge by examining the generalizability of risk factors for prison interpersonal violence within the cultural context of NI and by providing a clearer examination of the temporal relationship between these risk factors and involvement in prison interpersonal violence.

### Risk Factors for Prison Interpersonal Violence

Prison interpersonal violence is defined as the actual, attempted or threatened use of harm toward others in prison (Gadon et al., 2006). Self-report studies indicate that approximately one third of people engage in prison interpersonal violence, while a similar number report being victims of such violence (Bottoms, 1999; Braga et al., 2019; Edgar et al., 2003; Mears et al., 2013). Official measures can underestimate this behavior due to under-reporting and differential recording practices by prison staff (Bottoms, 1999; Braga et al., 2019; Byrne & Hummer, 2007). For instance, prison social norms may discourage people from reporting their victimization to prison staff and staff discretion and variations in perceptions regarding who is the aggressor may lead to variations in the reporting and recording of such incidents (Bottoms, 1999). One official measure of prison interpersonal violence is violent prison misconduct, which measures the extent to which individuals are found guilty of breaking prison rules by engaging in the actual, attempted, or threatened use of harm toward another. Sorensen and Cunningham (2010) state that roughly 10%–12% of all officially recorded rule infractions in prison are violent in nature. Yet, despite the shortcomings associated with official measures, these measures are often used to identify the causes and correlates of prison interpersonal violence and have been deemed to be valid measures of this behavior (Berg & DeLisi, 2006; Cunningham & Sorensen, 2007; Cunningham et al., 2011; Kuanliang et al., 2008; Steiner & Wooldredge, 2014; van Voorhis, 1994).

Studies have identified a number of risk factors for prison interpersonal violence. In line with the deprivation approach to understanding prison behavior, prison environmental factors have been identified as risk factors for prison interpersonal violence (Bottoms, 1999; Rocheleau, 2013; van der Laan & Eichelsheim, 2013). The deprivation approach emphasizes the role that prison environmental factors play in shaping behavior (Sykes, 1958). According to this approach, the prison environment deprives people of security, liberty, autonomy, heterosexual relationships, and goods and services (Sykes, 1958). Violence can be a means of responding to these deprivations, as violence can be used in self-defense, to deter victimization, deflect identity challenges and gain status (e.g., Butler, 2008; Edgar et al., 2003; Michalski, 2017; Rocheleau, 2013; Sykes, 1958). Self-report measures have been used to operationalize feelings of deprivation and safety among those in prison, alongside official measures of the provision of goods and services, prison security category, staffing levels and prison population characteristics (Bottoms, 1999; Rocheleau, 2013; Steiner et al., 2014; van der Laan & Eichelsheim, 2013). For example, self-reported feelings of deprivation and safety among inmates (Rocheleau, 2013; van der Laan & Eichelsheim, 2013), official measures of program availability and prison visitation (Blevins et al., 2010), prison security category (Bottoms, 1999), and a younger prison population (Cunningham et al., 2011) have been identified as risk factors for prison interpersonal violence.

Additional risk factors for prison interpersonal violence include staffing levels (Bierie, 2013), staff-prisoner relationships (Bottoms, 1999) and the extent to which people feel treated fairly (Bierie, 2013; Bottoms, 1999; Colvin, 1992). Research has found that harsh punishments, coercive disciplinary practices, and the use of inconsistent/unclear rules by prison staff can increase prison interpersonal violence and disorder (Colvin, 2007; Day et al., 2015; Dilulio, 1987). Similarly, others found a link between the submission of prison complaints and increased involvement in prison interpersonal violence (Bierie, 2013). Self-report measures are often used to assess people’s perceptions of staff-prisoner relationships, how prison rules are applied, as well as feelings of fairness (e.g., Day et al., 2015). Moreover, official measures of submitted prison complaints have been used to assess feelings of fairness (e.g., Bierie, 2013). These risk factors lend support to managerialist approaches, which also focus on prison environmental factors but stress the role that prison management and staff play in influencing behavior (Colvin, 1992; Dilulio, 1987).

Additional studies have identified individual characteristics and experiences as risk factors. Using both official and self-report measures to operationalize these variables, research has found that those who are younger are more likely to engage in violence (e.g., Cunningham & Sorensen, 2007; Day et al., 2015; Gaes et al., 2002; Kuanliang et al., 2008; Rocheleau, 2013; Woo et al., 2020), as well as gang members (Cunningham & Sorensen, 2007; Gaes et al., 2002; Kuanliang et al., 2008; Mears et al., 2013), people who have previously been imprisoned (e.g., Berg & DeLisi, 2006; Cunningham & Sorensen, 2007; Mears et al., 2013; Steiner & Wooldredge, 2009) and have a prior history of prison misconduct (Rocheleau, 2013). Further, reporting problematic alcohol and drug use (Steiner & Wooldredge, 2009; Woo et al., 2020), mental health issues (Glazener & Nakamura, 2020; Steiner & Wooldredge, 2009), coming from disadvantaged/deprived neighborhood (Mears et al., 2013), experiencing a brain/head injury (Matheson et al., 2020; Shiroma et al., 2010) and a persistent and/or violent criminal career (Cunningham & Sorensen, 2007; Kuanliang et al., 2008; Mears et al., 2013; Steiner & Wooldredge, 2009) are risk factors for prison interpersonal violence. The relationship between race and ethnicity with prison interpersonal violence is less clear, with some studies identifying race and ethnicity as possible risk factors (e.g., Berg & DeLisi, 2006; Gaes et al., 2002; Glazener & Nakamura, 2020; Steiner & Wooldredge, 2009) but other studies finding no significant relationship (e.g., Rocheleau, 2013; van der Laan & Eichelsheim, 2013).

Individual drug use while imprisoned has also been identified as a possible risk factor for prison interpersonal violence (e.g., Edgar et al., 2003; Friedmann et al., 2008). However, the few studies examining this relationship have either been qualitative in nature or tended to omit known risk factors for prison interpersonal violence (Edgar et al., 2003; Friedmann et al., 2008), raising questions as to whether prison drug use is a risk factor for prison interpersonal violence when controlling for other known risk factors. Additionally, Slade (2018) has argued that people in prison may engage in “dual harm” (i.e., harm to themselves and toward others) and that these individuals are especially likely to engage in misconduct (Slade, 2018). Indeed, research has found that men who self-harm in prison are at a 3.5-fold risk of prison interpersonal violence compared to those who do not (Slade et al., 2020). Yet, similar to research examining prison drug use, few studies have examined the possible relationship between self-harm in prison and prison interpersonal violence, and those that do have tended to omit known risk factors for such violence (Slade, 2018; Slade et al., 2020). Nevertheless, the identification of a number of individual characteristics and experiences as possible risk factors for prison interpersonal violence lends support to the importation approach to explaining prison behavior (Irwin & Cressey, 1962). The importation approach argues that it is the psychological characteristics, past experiences, attitudes, and beliefs people import into prison with them that are thought to be the most influential in shaping their behavior (Irwin & Cressey, 1962).

As the risk factors for prison interpersonal violence support the deprivation, managerial and importation approaches to understanding prison behavior, efforts have been made to integrate these approaches (e.g., Blevins et al., 2010; van der Laan & Eichelsheim, 2013; Wooldredge, 2020). Using general strain theory, it has been argued that prison conditions (highlighted by the deprivation and managerialist approaches) may act as a source of strain, with individual characteristics influencing how people adapt to this strain and the coping strategies they employ (Blevins et al., 2010; Wooldredge, 2020). General strain theory proposes that people are usually compliant but may engage in rule breaking behavior in response to strain (Agnew, 2001). Three main sources of strain include exposure to negative stimuli, when positively valued stimuli are removed or when individuals are unable to achieve a desired goal (Agnew, 2001). Prison interpersonal violence is believed to occur when people experience strain in prison and when their individual characteristics, experiences and beliefs lead them to use violence as a means of responding to this strain (Blevins et al., 2010).

## The Present Study

The present study explains whether previously identified risk factors for prison interpersonal violence can predict violent prison misconduct in NI. In comparison to other jurisdictions, NI has a small prison population, consisting of only four prisons (two for adult males, one for females and one for young males) and a lower rate of imprisonment (ICPR, 2020; NIPS, 2019). Similar to other jurisdictions, it is overwhelming adult males that are imprisoned, although a slightly higher percentage of foreigners and those on pre-trial detention/remand can be found compared to other jurisdictions, such as America (ICPR, 2020). Life in NI prisons is also comparable to other developed, Western, democratic jurisdictions in terms of its conditions, regime, and management (Butler, 2016). Yet, NI’s history of conflict has meant that nationality and religion have played a key role in shaping identity, diversity, and equality more so than may be experienced elsewhere (Harvey, 2012; O'Dowd et al., 1980). This conflict affected NI prisons with paramilitary prisoners becoming the main “gangs” that NI prison staff must manage, posing particular security challenges for prison staff and management (Butler, 2016, 2020; Butler et al., 2018).

By using a prospective longitudinal research design to examine whether previously identified risk factors for prison interpersonal violence can predict violent prison misconduct in NI, the present study enhances our understanding in three ways. Firstly, it examines the robustness and validity of previously identified risk factors for prison interpersonal violence by investigating their applicability to NI. Secondly, by using a prospective longitudinal research design, this study clarifies the temporal relationships involved by investigating whether these risk factors can predict future involvement in prison interpersonal violence. Thirdly, the influence of prison drug use and self-harm in prison on violent prison misconduct will be quantitatively examined while controlling for other known risk factors.

## Methodology

### Data

The study utilized longitudinal administrative data from the prison service records of imprisoned adult males detained in Maghaberry Prison. The sample consists of 429 adult males imprisoned in Maghaberry Prison on November 22, 2017, who continued to be imprisoned 1 year later on November 22, 2018. There are only two adult male prisons in the Northern Ireland Prison Service (NIPS), Maghaberry Prison and Magilligan Prison. Maghaberry Prison is the larger of the two prisons and holds all high security, remand, and separated prisoners (NIPS, 2019). The term “separated prisoners” refers to those paramilitary prisoners who claim that their offences are politically motivated and demand to be held separately to the rest of the prison population (Butler, 2020). Of the 429 adult males in this study, 23 were separated prisoners. Information on those detained in Magilligan prison on November 22, 2017 was not available to the research team; however, any detainee that transferred to Magilligan prison during the follow-up period was included in the sample.

Furthermore, a minority of the sample (*n* = 98, 23%) were not imprisoned throughout the follow-up period but had been released and subsequently reimprisoned. All adult males are first imprisoned in Maghaberry Prison before potentially being transferred to Magilligan Prison, which means the sampling method provided a relatively representative view of the NI population of imprisoned adult males in the NIPS at the two time points. The sample of 429 adult males represents approximately 33% of the total average daily population of adult males detained in NIPS during 2017/2018 (NISRA, 2020). The data comes from anonymized administrative records that provided measures on violent prison misconduct and a number of risk factors linked to violent prison misconduct in previous research. Measures on the changing nature of prison conditions, staffing levels, and attitudes and experiences, was outside the scope of the dataset. Nevertheless, administrative data can provide an exact record of misconduct, offence history, prison drug tests passed, etc., that otherwise risk recall bias. Additionally, administrative data has proven to provide valid rich data and is frequently used to examine the causes and correlates of prison interpersonal violence (Berg & DeLisi, 2006; Cunningham & Sorensen, 2007; Cunningham et al., 2011; Kuanliang et al., 2008; Steiner & Wooldredge, 2014; van Hoorhis, 1994).

### Measures

#### Dependent

*Violent prison misconduct.* This refers to the number of occasions individuals were found guilty of engaging in violent prison misconduct during the 1-year follow-up period. Behaviors were recorded as violent prison misconduct if they involved committing an assault on another person (inmate or staff) and/or behaviors that endangers others, such as fighting/wrestling or piercing with a needle or other implements.

#### Independent

To investigate what explains violent prison misconduct, a number of measures were included to capture the risk factors identified in previous research. Notably, all the independent measures were pulled from the administrative records at time point 1 and refer to the participants’ characteristics on the exact date of November 22, 2017.

*Age.* Participants’ age in years.

*Race.* The small number of non-White ethnic/racial participants in NI prisons limited distinguishing between different groups. Participants’ were recorded into “White” and “non-White” based on reported race/ethnicity. NI recognizes Travelers as a distinct racial group under the Race Relations (Northern Ireland) Order 1997 and were therefore included in the “non-White” category, along with those reporting other non-White racial identities.

*Nationality.* Nationality is a key marker of diversity and identity, especially in NI, and can influence the attitudes people hold toward state officials. For instance, people in NI who identity as “Irish” can hold more negative attitudes toward criminal justice officials and be less likely to work in these organizations compared to those who identify as “British” (Deloitte, 2016; Ellison & Smyth, 2000; O'Dowd et al., 1980). Nationality was captured from participants self-identifying in the records as one of the following four categories: “Irish,” “British,” “Northern Ireland,” or “Other nationality.”

*Religion.* Religion is another key marker of diversity and identity in NI. During the NI conflict, people could be treated differently depending on their religious identity (Harvey, 2012). Consequently, religion in NI is used to reflect identity rather than religiosity. Self-reported religion was recoded into three categories: “Catholic,” “Protestant,” or “Other religion.”

*Neighborhood deprivation.* The postcode of participants’ address prior to their incarceration was detailed in the dataset. If participants had a NI postcode, it was possible to match the postcode to the NI Multiple Deprivation Measures 2017 (NISRA, 2017) to obtain a measure of neighborhood deprivation. Neighborhoods in NI are broken up into 890 small areas; the deprivation measured ranks these areas from 1–890 (NISRA, 2017). Of the 429 participants, 355 had NI postcode, while 75 participants did not have a NI postcode because they resided in a different jurisdiction (*n* = 13, 3%), were of no fixed abode prior to their imprisonment (*n* = 45, 10%) or their address was unknown (*n* = 17, 4%). As these 75 participants were not random but a diverse group, observations from these cases were imputed from the average deprivation rank and a separate dummy variable was included to indicate that these individuals were missing. Preliminary analysis comparing the robustness of the model to excluding these participants showed no notable differences. The rankings were reverse coded for ease of interpretation, with higher values indicating higher levels of deprivation.

*Medical history.* Additionally, the dataset captured information about the self-declared medical history of participants, recoded into six measures indicating a history of: mental health issues; head injury/epilepsy; behavioral issues; impairments (including communication, hearing, speech and/or vision impairments); addiction and self-harm. Each measure was recoded into a dummy variable indicating “Yes” or “No” on whether participants had disclosed a history of experiencing that issue on committal.

*Offence history.* Participants’ history of committing certain types of criminal offences were collapsed into four separate domains: violence, property, drugs, or other offences. These measures were not mutually exclusive and were dummy coded, with “Yes” or “No” indicating whether participants had a history of committing that particular offence.

*Separated status.* Unlike in the USA and other jurisdictions, the main “gangs” operating inside and outside prison in NI are paramilitary groups who are delineated on political and religious lines (Butler et al., 2018). Previous research has indicated that involvement in gangs can predict violent prison misconduct (Gaes et al., 2002; Mears et al., 2013; Steiner et al., 2014). Upon entering the NIPS, members of paramilitary groups can claim “separated” status, whereby they seek recognition that their offences are politically motivated and demand to be held separately to the rest of the prison population, forming roughly 4% of the prison population (Butler, 2020). Separated status was coded as a dummy variable, with “yes” indicating separated status and “no” indicating that the participant did not have separated status.

*Prison complaints.* The number of official complaints that participants had submitted to NIPS throughout their time in custody up until November 22, 2017 was contained in the dataset. To take into account variations in time spent imprisoned, the number of complaints submitted was divided by the total days participants had been imprisoned.

*Prison visits.* Information on prison visitation was captured in the dataset. To again take account of variations in time spent imprisoned, the total number of visits participants had received was divided by the total days participants had been imprisoned.

*Percentage prison drug tests passed.* The percentage of prison drug tests participants had successfully passed up until November 22, 2017 was also included in the anonymized NIPS administrative dataset. This measure provides an indication of drug use in prison; however, it should be noted that prison drug tests were not administered during the first 30 consecutive days of an individual’s imprisonment. Accordingly, participants who had not yet taken a drug test were generally those who were imprisoned for the first time and/or had not yet served 30 consecutive days of imprisonment. These participants (*n* = 17, 4%) were coded as 100% passed and are identified in the measure below. Preliminary analysis excluding this group showed no notable changes in the analysis.

*First 30 days in prison.* To identify those who had not yet been imprisoned for 30 consecutive days, a dummy measure of “Yes” and “No” was used. Previous studies suggest individuals who are in the early days of their imprisonment may be especially at risk of violent prison misconduct (Edgar et al., 2003; Sykes, 1958).

*Number of Supporting Prisoners at Risk (SPAR) referrals.* The dataset also contained information on the number of times individuals were referred under the SPAR policy. Referrals under this policy are made if people have engaged in, or staff are concerned that they are very likely to engage in, serious incidents of self-harm and/or attempt to take their own life (Sudgen, 2016). To account for differences in time spent imprisoned, the total number of SPAR referrals was divided by the total days participants had been imprisoned.

*Past involvement in prison misconduct.* Moreover, the dataset contained a measure of all past involvement in misconduct. Due to the nature of the dataset, it was not possible to separate out prior nonviolent and violent prison misconduct. To again take account of differences in time spent imprisoned, the total number of times participants were found guilty of committing prison misconduct was divided by the total days they had spent imprisoned.

*Periods of incarceration.* The total number of occasions that participants had been incarcerated within the NIPS was recorded in the dataset as a continuous variable.

#### Control

*Days spent imprisoned during the follow-up period.* Participants may have been released and reimprisoned during the 1-year follow-up period, affecting the opportunities they had to engage in violent prison misconduct. As such, a continuous measure of the number of days participants had spent imprisoned between November 22, 2017 and November 22, 2018 was included, using the Stata command exposure (which adjusts the model accordingly and sets the covariate to 1).

### Procedure

Ethical approval to conduct the research was obtained from the NIPS and Queen’s University Belfast (QUB), with the NIPS approving access to the anonymized NIPS administrative dataset. Discussions were held between NIPS management and the QUB researchers regarding the information routinely captured by the NIPS Prison Records Information Management System (PRISM) to identify variables of interest to the present study. It was agreed that an anonymized dataset containing the variables of interest would be provided to the researchers for analysis, with the results being used to inform the development of NIPS policies and practices. This dataset was generated by taking a “snapshot” of all those imprisoned in Maghaberry prison on November 22, 2017 and a member of NIPS prison staff was assigned to work with the QUB researchers to collate the information from PRISM into an anonymized dataset, which ensured that only NIPS personnel had access to the unanonymized dataset. The anonymized dataset was provided to the QUB researchers in an excel file format, with this data being cleaned, coded, and entered into STATA version 15 for analysis. Any queries that emerged during this process were resolved through discussions with the NIPS member of staff, or NIPS management, when necessary.

### Analytic Strategy

Using the countfit command in Stata to conduct diagnostic tests revealed that a negative binominal regression best fit the data. A negative binominal regression analysis is a specific type of regression analysis for count data used when the outcome event occurs infrequently. In this research, the outcome event was violent prison misconduct, which was only committed by 58 of the 429 participants (*n* = 14%). Preliminary analysis confirmed that the assumptions for conducting this regression analysis were met. One imprisoned male was excluded from the analytic sample (not in the *n* = 429) due to having a value of 12 on the dependent variable, which was in excess of the next-highest value of six. A single observation set apart from the other values can led to unstable estimates in maximum likelihood equations (Agresti, 2018). Preliminary analysis confirmed this was a unique case and excluding the case made mostly minor changes in the model around the *p* < .10 level. However, the measure of first 30 days in prison lost significance with the exclusion of this case, from previously being significant at the *p* < .001 level. This was explained by this case being one of only 17 participants that were in the first 30 days of their imprisonment on November 22, 2017. Diagnostic tests showed no issues with multicollinearity. As previously described, the number of days spent imprisoned during the 1-year follow-up was included in the analysis using the exposure command in Stata to adjust for varying times spent imprisoned during the follow-up period.

## Results

Table 1 presents the descriptive statistics. The average age is 36 and 5% reported a non-White race. Over two-thirds claimed NI nationality, compared to 10% Irish, 14% British, and 7% other. The largest religious identity was Catholic (51%), followed by Protestant (36%), and other religion (13%). The average deprivation rank was 613 with a range from 7 to 888, while 17% of participants did not have a deprivation measure as they lacked a NI postcode. Among the medical histories disclosed, self-harm (57%) and addiction (52%) were the most common, followed by mental health issues (40%), head injury/epilepsy (15%), impairments (10%), and behavioral issues (5%). Nearly all participants (91%) had a committed a violent offence, while just over half (51%) had committed a property offense. Over a quarter (28%) had committed a drug offence and 35% had committed another type of offense, not captured in the violence, property, or drug categories. Only 5% of participants had claimed separated status. Taking into account the varying time spent in NI prisons, participants averaged 0.016 complaints, 0.070 prison visits, 0.005 SPAR referrals, and 0.006 incidents of prison misconduct per day. On average participants had passed 78% of prison drug tests, although 4% had not yet taken a prison drug test. Participants had been incarcerated on average 5.6 times and, during the 1-year follow-up, the average amount of time participants spent imprisoned in the NIPS was 338 days.

**Table 1.** Descriptive Statistics.

Measures	Mean	(*SD*)	min	max	%
Violent prison misconduct	0.280	(0.873)	0	6	
Age	35.501	(10.629)	21	89	
Non-White					5.13
Nationality					
Northern Ireland					68.07
Irish					10.26
British					14.45
Other					7.23
Religion					
Catholic					51.05
Protestant					35.90
Other religion					13.05
Deprivation	613.009	(224.711)	7	888	
Missing area flag					17.48
Medical history					
Mental health					40.33
Head injury/epilepsy					15.38
Behavioral issues					4.66
Impairments					9.56
Addiction					51.98
Self-harm					57.11
Offence history					
Violence					90.91
Property					54.55
Drugs					28.21
Other					34.50
Separated status					5.36
Prison complaints	0.016	(0.067)	0	0.678	
Prison visits	0.070	(0.105)	0	1.516	
Percentage prison drug tests passed	0.776	(.254)	0	1	
First 30 days in prison					3.73
Number of SPAR referrals	0.005	(0.033)	0	0.667	
Past involvement in prison misconduct	0.006	(0.011)	0	0.119	
Periods of incarceration	5.592	(5.589)	1	44	
Total days in prison during follow-up	338.187	(64.770)	35	365	

Table 2 presents the negative binominal regression to examine if previously identified risk factors for prison interpersonal violence predicted violent prison misconduct in NI during the 1-year follow-up. Those who identified as Irish had a higher risk of violent prison misconduct compared to those with a NI national identity (*B* = 1.042; *p* < .01). Those classified as other religion had lower risk of a violent prison misconduct than Catholics, with marginal significance (*p* = .089). Deprivation had a negative relationship with violent prison conduct (*B* = –0.001; *p* < .05), with those coming from more deprived areas being less likely to commit violent prison misconduct. Additionally, those without a NI postcode had a lower rate of violent prison misconduct compared to those with a NI postcode (*B* = –1.210; *p* < .01). In regards to medical history, those reporting a history of addiction had a lower rate of violent prison misconduct compared to those who did not report a history of addiction (*B* = –1.230; *p* < .001). Further, those reporting a history of head injury/epilepsy had an increased rate of violent prison misconduct compared to those that did not report this condition, but at the marginal significance level (*p* = .063).

**Table 2.** Negative Binominal Regression for Violent Prison Misconduct.

Measures	*B*			*SE*		95% CI
Age	–0.023			0.020		[–0.061, 0.015]
Non-White (ref = White)	–0.257			0.720		[–1.668, 1.154]
Nationality (ref = Northern Ireland)						
Irish	1.042	**		0.375		[0.306, 1.777]
British	0.594			0.423		[–0.235, 1.422]
Other	–12.671			485.796		[–964.813, 939.470]
Religion (ref = Catholic)						
Protestant	–0.406			0.393		[–1.177, 0.365]
Other religion	–1.037	t		0.610		[–2.233, 0.159]
Deprivation	–0.001	*		0.001		[–0.026, 0.000]
Deprivation missing (ref = has postcode)	–1.210	**		0.413		[–2.020, –0.400]
Medical history (ref = does not report this history)						
Mental health	0.155			0.312		[–0.456, 0.766]
Head injury/epilepsy	0.513	t		0.276		[–0.029, 1.054]
Behavioral issues	–0.717			0.551		[–1.796, 0.363]
Impairments	0.506			0.366		[–0.211, 1.224]
Addiction	–1.230	***		0.347		[–1.910, –0.551]
Self-harm	0.393			0.372		[–0.337, 1.122]
Offence history (ref = no history of committing this offence)						
Violence	–0.108			0.525		[–1.136, 0.921]
Property	0.685	t		0.390		[–0.079, 1.450]
Drugs	–0.035			0.301		[–0.626, 0.556]
Other	–0.158			0.288		[–0.723, 0.406]
Separated status (ref = no)	–4.576			3.389		[–11.219, 2.067]
Prison complaints	12.322	*		5.755		[1.041, 23.602]
Prison visits	–5.707	t		3.391		[–12.354, 0.940]
Percentage prison drug tests passed	–0.740			0.610		[–1.936, 0.455]
First 30 days in prison (ref = no)	0.205			1.641		[–3.011, 3.420]
Number of SPAR referrals	2.554			4.707		[–6.673, 11.779]
Past involvement in prison misconduct	56.975	***		8.419		[40.474, 73.475]
Periods of incarceration	0.122	***		0.024		[0.075, 0.169]
Constant	–6.505	***		1.091		[–8.652, –4.375]
Log likelihood	–178.689					
Likelihood ratio *χ*^2^	151.33***					
*df*	27					
*n*	429					

*Note*. ^t^*p* < .10; *p < .05; **p < .01; ***p < .001.

In relation to factors that relate specifically to crime and imprisonment, those with a history of property offenses had an increased rate of violent prison misconduct compared to those without a history of property offences, but this relationship was only marginally significant (*p* = .079). Prison complaints were also significantly related to violent prison misconduct, with those who submitted more complaints being at a higher rate of violent prison misconduct (*B* = 12.322; *p* < .05). Prison visitation was negatively associated with violent prison misconduct, with more prison visits decreasing the risk of violent prison misconduct, but only at the marginal significance level (*p* = .092). Past involvement in prison misconduct showed a strong relationship with violent prison misconduct, with those reporting a greater involvement in past misconduct being at an increased rate of violent prison misconduct during the 1-year follow-up (*B* = 56.975; *p* < .001). Furthermore, the number of times participants were incarcerated was related to violent prison misconduct, with individuals who were incarcerated more often demonstrating an increased rate of violent prison misconduct during the 1-year follow-up (*B* = 0.122; *p* < .001).

## Discussion

In answer to the study’s research question, only a small number of previously identified risk factors for prison interpersonal violence were found to be significant in the NI context. Only nationality, deprivation, addiction, prison complaints, past involvement in prison misconduct and periods of incarceration reached statistical significance, with religion, head injury/epilepsy, property offences and prison visits significant at the marginal level. Of particular note is that nationality and religion emerged as predictors of this behavior. As previously stated, nationality and religion are key markers of identity in NI, with the history of NI indicating that people could be treated differently depending on their religion, and the NI conflict occurring due to this unequal treatment and differing views on nationality (Harvey, 2012; O'Dowd et al., 1980). Unlike in other jurisdictions where race and ethnicity may be especially important for shaping identity, diversity, and experiences of conflict and equality, the cultural context of NI has placed a greater emphasize on nationality and religion.

Similarly, the main “gangs” operating in and outside of NI prisons are paramilitary-affiliated and delineated based upon religion and nationality (Butler et al., 2018). Paramilitary groups are involved in violence and criminality (Hogg & Butler, 2018; Hourigan et al., 2018). However, within NI prisons they have primarily focused on demanding separated conditions, political recognition of their offences and undermining the legitimacy of the State rather than using violence to dominate the prison population or illegal prison markets (Butler, 2020; Butler et al., 2018). This history, along with the small group size, may explain why no relationship was found, in contrast to research in other jurisdictions that links gang membership to prison interpersonal violence (Gaes et al., 2002; Mears et al., 2013; Steiner et al., 2014). This result, combined with the significance of nationality and religion, suggests that cultural context matters when attempting to generalize the potential causes and correlates of this behavior from one jurisdiction to another.

While a significant relationship was observed between deprivation and violent prison misconduct, it was unexpected in that those who came from more deprived areas were less likely to commit violent prison misconduct. One possible explanation may be that individuals from less deprived areas felt more vulnerable to victimization and engaged in violence as a means of deterring others from victimizing them. Past studies have found that imprisoned men deliberately engage in prison interpersonal violence to project an identity of strength, gain status among the prisoner social hierarchy and deter victimization (e.g., Butler, 2008; Edgar et al., 2003; Michalski, 2017). The finding that those without a NI postcode were significantly less likely to commit violent prison misconduct may reflect the tendency for foreign prisoners to experience more isolation and less engagement with prison life due to their specific needs (Barnoux & Wood, 2013). However, further research is needed to explore these possible explanations and examine if these findings are unique to NI.

Prison drug use and serious self-harm were not found to predict violent prison misconduct. However, past involvement in misconduct and prior incarceration significantly predicted violent prison misconduct, indicating that those with a history of offending behavior were more likely to engage in violence and were less deterred from this activity, given that previous punishments had failed to deter them from continued involvement in this behavior. A history of violent offences was not significant, possibly because 91% of participants reported a history of violent offences limiting its utility in distinguishing between those who engaged in violent prison misconduct and those who did not. While further research is needed to explore the link between a history of property offences and violent prison misconduct, it is possible that if people continued to engage in property offences while imprisoned, they may become involved in violence as past studies have found that cell theft often results in prison interpersonal violence (Edgar et al., 2003). A history of head injury/epilepsy may increase the risk of prison interpersonal violence due to difficulties in processing emotions and cognitions, as well as monitoring and controlling behavior (Matheson et al., 2020; Shiroma et al., 2010). A history of addiction was also found to reduce the risk of violent prison misconduct, possibly reflecting a tendency to cope with stressful situations through withdrawal or escapism. Future research could explore this finding further.

Similar to previous research, prison complaints were found to increase the risk of violent prison misconduct (Bierie, 2013), while prison visits were only marginally significant. As in past studies, those who felt treated unfairly were more likely to engage in violence (Bierie, 2013; Bottoms, 1999; Colvin, 1992). Previous research suggests that visitation may reduce misconduct by ameliorating the deprivations associated with imprisonment and by visitors exerting social control over those imprisoned, although its effects are believed to be too short-term to create lasting improvements in behavior (Siennick et al., 2013). Questions also remain over the relationship between visitation and violent prison misconduct as prison incentive schemes tend to allocate extra visits to those who comply with prison rules. More research is needed to examine if this relationship reflects a tendency for compliant people to be allowed extra visits or the ability of visitation to reduce misconduct through social control and the amelioration of the deprivations associated with imprisonment.

The risk factors for violent prison misconduct in NI provides the strongest support for the role of individualistic characteristics, in line with the importation theory. At the same time, due to the nature of the administrative data, the inclusion of measures capturing managerialist, environmental and coping factors were limited and should be considered in future studies. Furthermore, the findings suggest drug use and self-harm in prison did not predict violent misconduct, contradicting previous research (Edgar et al., 2003; Friedmann et al., 2008; Slade, 2018; Slade et al., 2020).

Of course, there are limitations associated with this research. Official measures tend to underestimate the prevalence of prison interpersonal violence and can be influenced by staff discretion and differential recording practices (Bottoms, 1999; Braga et al., 2019; Byrne & Hummer, 2007). Equally, the sample of adult males and cultural context of NI may limit the generalizability of the findings to other groups and jurisdictions. The inclusion of additional measures of prison environmental factors (such as access to goods and services, feelings of safety, deprivation, etc.), was outside this study’s scope and may provide fruitful lines of study for future research. Lastly, the small group size on some measures (e.g., first 30 days in prison, impairment, behavioral issues, head injury/epilepsy and separated status) may limit the ability of the analysis to detect a relationship between these measures and violent prison misconduct. Nevertheless, the sampling of administrative data allowed for inclusion of official data that had been entered into the system going back decades, and it allowed a complete snapshot sample of every adult male imprisoned in Maghaberry Prison, which is the largest and most diverse of the two adult male prisons in NI. In other words, the analytic sample was very close to capturing the full prison population of adult males in NI that were incarcerated over the two time points. Furthermore, the prospective longitudinal research design is a key strength and by expanding this research to NI the study offers insights into the exportability of research on the risk factors of prison interpersonal violence to different jurisdictions.

Finally, based on these findings, suggestions for policy and practice include expanding risk assessment for prison interpersonal violence to incorporate a consideration of the rate at which people have previously engaged in prison misconduct, submitted prison complaints, received visits, and previously been imprisoned. These factors should be considered in addition to individual factors, such as prior criminal offences, medical history, demographic factors, and neighborhood deprivation. Interventions should seek to improve feelings of fairness in the prison regime and interactions with prison staff, as well as identify and treat underlying medical issues, such as head injury/epilepsy. Interventions seeking to support prison visitation and rebuild relationships between those imprisoned and their loved ones may also prove useful. Lastly, the reliance on deterrence as a means of deterring rule infractions may need to be re-examined given that those who had previously been imprisoned and punished through the prison disciplinary system were at an increased risk of engaging in violent prison misconduct. A more effective strategy for reducing prison interpersonal violence may be to provide specialist services and supports to address the factors believed to be contributing to their violent behavior.

## Author Biographies

**Michelle Butler**, PhD, is a senior lecturer in criminology in the School of Social Sciences, Education and Social Work, Queen’s University Belfast. Her research focuses on imprisonment and emphasizes improving people’s outcomes through collaborative, multidisciplinary efforts. She is also interested in how imprisonment may affect the families of those imprisoned and the achievement of the UN Sustainable Development Goals.

**Catherine B. McNamee**, PhD, is a lecturer in sociology in the School of Social Sciences, Education and Social Work, Queen’s University Belfast. Her research focuses on quantitative methods, demography, and race–ethnicity.

**Dominic Kelly**, MSSc, is employed within psychology services in the Northern Ireland Prison Service. His research focuses on imprisonment, paying specific attention to the working experiences of prison staff and staff support mechanisms.
